# Comparative treatment outcomes of a single long stent vs. overlapped short stents in acute myocardial infarction

**DOI:** 10.3389/fcvm.2023.1284396

**Published:** 2023-12-19

**Authors:** Doo Hwan Lee, Seok Oh, Min Chul Kim, Doo Sun Sim, Young Joon Hong, Ju Han Kim, Youngkeun Ahn, Jae Bok Han, In Soo Kim, Myung Ho Jeong

**Affiliations:** ^1^ Department of Cardiology, Chonnam National University Hospital, Gwangju, Republic of Korea; ^2^ The Cardiovascular Convergence Research Center of Chonnam National University Hospital Nominated by Korea Ministry for Health and Welfare, Gwangju, Republic of Korea; ^3^ Department of Radiological Science, Dongshin University, Naju, Republic of Korea; ^4^ Department of Cardiology, Chonnam National University Medical School, Hwasun, Republic of Korea

**Keywords:** comparative study, coronary intervention, stents, myocardial infarction, percutaneous coronary intervention

## Abstract

**Objectives:**

There is no consensus regarding the optimal choice between single long stent (SLS) and overlapped double short stents (DSS) in patients with acute myocardial infarction (AMI). Therefore, we aimed to compare treatment outcomes among patients with AMI treated with these two different stenting methods.

**Methods:**

In total, 537 patients with AMI from a single tertiary center were categorized into two groups: (1) those who received an SLS (stent length ≥38 mm) (*n* = 254; 47.3%) and (2) those who received overlapped DSS (individual stent lengths <38 mm) (*n* = 283; 52.7%). The primary outcome was the incidence of major adverse cardiac and cerebrovascular events (MACCEs) within 1 year.

**Results:**

The mean age of participants was 65.4 years, and 75.0% were male. Patients receiving an SLS had a higher rate of serum creatinine level ≥1.5 mg/dl (16.3% vs. 8.9%, *p* = 0.009) but a lower rate of hypertension (46.8% vs. 55.8%, *p* = 0.038), lesser total stent length (38.26 ± 1.31 vs. 45.20 ± 9.25 mm, *p* < 0.001), total procedure time (41.40 ± 15.74 vs. 53.31 ± 21.75 min, *p* < 0.001) and total contrast volume (134.13 ± 30.72 vs. 160.57 ± 39.77 ml, *p* < 0.001) than in those receiving DSS. One-year MACCEs were comparable between the two groups before [hazard ratio (HR), 1.33; 95% confidence interval (CI), 0.80–2.24] and after adjusting for covariates (HR, 1.21; 95% CI, 0.67–2.19).

**Conclusions:**

Stenting with an SLS demonstrated similar outcomes compared to those achieved when using stenting with overlapped DSS in patients with AMI. Therefore, if the deliverability is acceptable, stenting with an SLS appears to be a safe and effective strategy for AMI treatment.

## 1. Introduction

In the modern age of percutaneous coronary intervention (PCI), many interventional cardiologists routinely encounter challenging cases of coronary artery disease (CAD) with a wide variety of complex lesions, thereby making it difficult to decide whether to implant multiple stents or a single long stent (SLS). Certain types of CAD involve extended or bifurcated coronary lesions that cannot be managed with implantation of a single stent; therefore, they tend to be treated with multiple stents (1). However, despite the widespread utilization of multiple stents, their implantation is associated with a greater risk of stent thrombosis or restenosis post-PCI (2, 3). Although newer-generation multiple drug-eluting stents (DESs) appear to provide favorable safety outcomes comparable to those of a single DES (4), these results have remained controversial (5, 6).

Among CAD, acute myocardial infarction (AMI) is an emergent medical illness that requires urgent intervention and thus necessitates well-timed and effective revascularization of the infarct-related artery (IRA) (7). In clinical settings of AMI, the primary operator must make a prompt decision regarding the stent implantation strategy during PCI, and the choice between the two stenting methods (multiple stents vs. a single stent) is challenging. However, there is a distinct lack of clinical evidence regarding the outcomes in patients with AMI treated with SLS vs. overlapped double short stents (DSS). To bridge this gap, the present study sought to examine the differences in clinical characteristics and treatment outcomes in patients with AMI receiving SLS vs. overlapped DSS.

## 2. Methods

### 2.1. Study design

**Figure 1:**
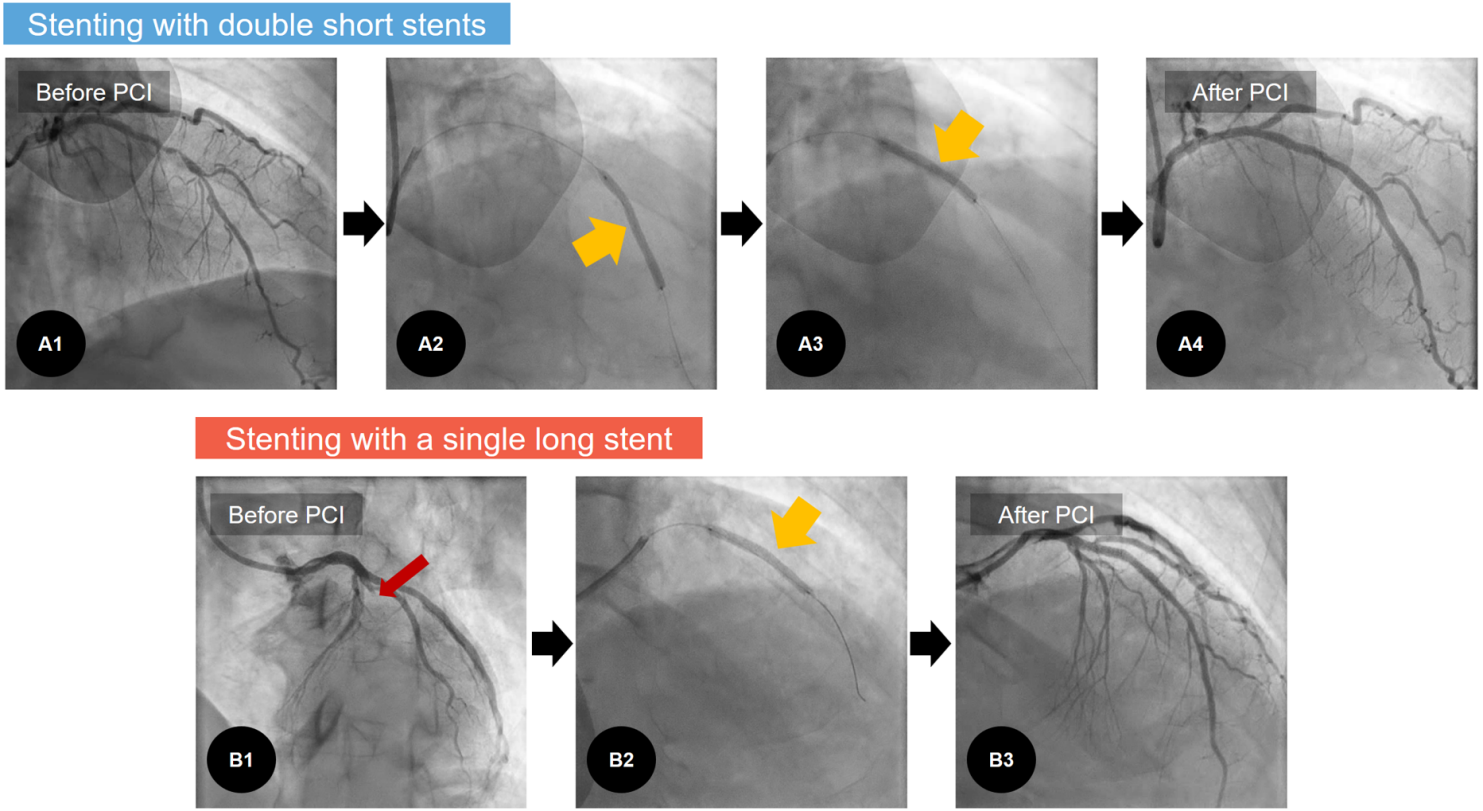
Representative examples of coronary stenting: stenting with double short stents. (**A**) Versus with a single long stent. (**B**) A1: in one case of AMI, diffuse coronary stenosis is noted in LAD, A2–3: With the overlapping stent technique, two short DESs (individual stent lengths <38 mm) are implanted at middle and distal parts of the LAD (yellowish arrows). A4: Post-PCI angiogram revealed good angiographic results. B1: In another case of AMI, there is 100% occlusion in the LAD (red arrow). B2: To treat this, a single long DES (stent length ≥38 mm) is implanted. B3: Thereafter, post-PCI angiogram reveals good angiographic results. AMI, acute myocardial infarction; DES, drug-eluting stent; LAD, left anterior descending coronary artery; PCI, percutaneous coronary intervention.

**Figure 2:**
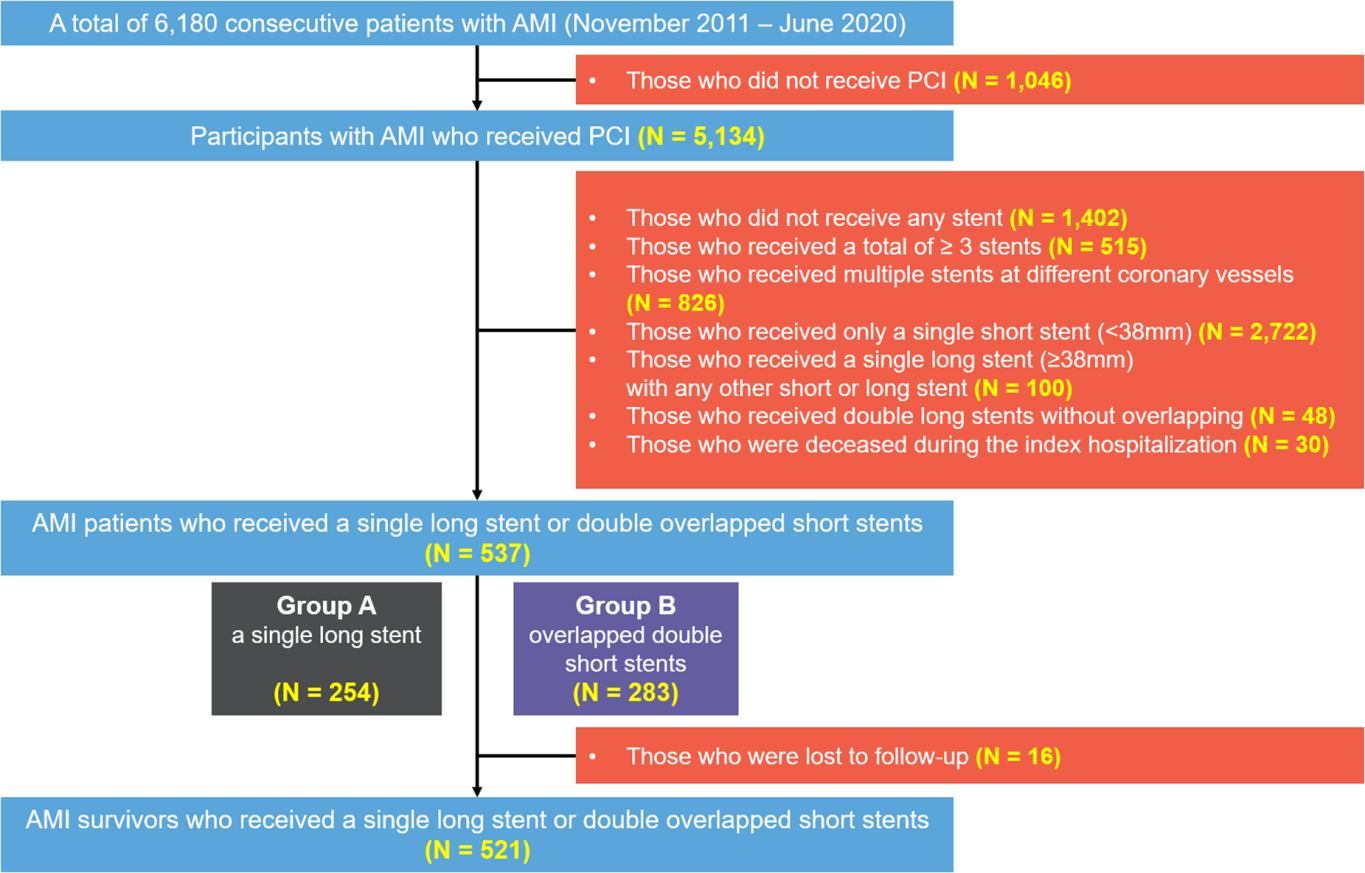
Flowchart of the present study. AMI, acute myocardial infarction; PCI, percutaneous coronary intervention.

This study is based on a non-randomized retrospective analysis in Chonnam National University Hospital (CNUH), a single tertiary cardiovascular hospital located in Gwangju, Republic of Korea. All clinical data were collected from patients with AMI undergoing PCI with 1-year clinical follow-up at CNUH. From November 2011 to June 2020, a total of 6,180 patients with AMI were initially screened. Patients who had not undergone PCI were excluded, yielding 5,134 patients. The following patients were then excluded: (1) not receiving any stent, (2) with ≥3 stents, (3) with multiple stents for different coronary vessels, (4) receiving only a single short stent, (5) receiving an SLS with any other short or long stent, (6) receiving DSS without overlapping, and (7) who were deceased during the index hospitalization. This process yielded 537 consecutive patients with AMI who were enrolled and categorized into two groups depending on the stenting method: patients in group A underwent PCI with only SLS (stent length ≥38 mm) (*n* = 254), and those in group B underwent PCI with overlapped DSS (individual stent lengths <38 mm) (*n* = 283). Representative examples are illustrated in Figure 1. All stenting procedures were performed to the IRA lesions. To compare the treatment outcomes following AMI, we excluded a total of 16 patients who were lost to follow-up from the survival analysis. Finally, the treatment outcomes in 521 consecutive survivors were analyzed. The study scheme is illustrated in Figure 2.

### 2.2. Definitions

As stated in several contemporary guidelines (7), AMI refers to an increase or decrease in cardiac biomarkers and associated clinical indicators, including one or more of the following conditions: (1) ischemia-driven clinical symptoms and/or signs; (2) newly identified changes on an electrocardiogram indicative of myocardial ischemia, including ST-segment deviation, T-wave inversion, or new detection of pathological Q-waves; (3) definite evidence suggesting loss of viable myocardium or regional wall motion abnormalities visualized with any cardiovascular imaging tool; and (4) presence of intracoronary thrombus during coronary angiography. Among AMI, ST-segment elevation myocardial infarction (STEMI) is a medical condition with overlapping components of the AMI definition with new-onset ST-segment elevation in at least two continuous leads (7).

Imaging guidance during the index PCI relates to adjunctive usage of any intracoronary imaging tool, such as intravascular ultrasound (IVUS) or optical coherence tomography (OCT), to evaluate intracoronary lesion characteristics. An IRA is an AMI-responsible coronary vessel that is obstructed or narrowed by atherothrombosis. Left main coronary artery (LMCA) disease refers to an LMCA lesion with a diameter stenosis ≥50%. Multivessel CAD was defined by ≥70% diameter stenosis in ≥2 coronary arteries or ≥70% stenosis in one coronary artery with LMCA disease. Coronary lesion characteristics within an IRA were stratified based on angiographic findings in consonance with the American College of Cardiology/American Heart Association (ACC/AHA) lesion complexity system (8). The antegrade intracoronary flow was stratified according to the thrombolysis in myocardial infarction (TIMI) flow grading system (9). To evaluate left ventricular systolic function, the left ventricular ejection fraction (LVEF) was assessed using a two-dimensional transthoracic echocardiogram. Peak troponin-I level was defined as the highest level of troponin-I measured within 72 h after hospital admission.

Meanwhile, we also investigated results from quantitative coronary angiography (QCA) in all study participants. These were derived from artificial intelligence-based automated QCA by a novel software (MPXA-2000, Medipixel) using a deep learning algorithm to segment and analyze angiogram images. Data on QCA included lesion length, mean/proximal/distal reference vessel diameter, minimum lumen diameter, and percent diameter stenosis.

As mentioned earlier, a long stent was defined based on a stent length ≥38 mm, whereas a short stent was defined based on a stent length <38 mm. In other words, DSS were defined as individual stents <38 mm in length (i.e., two short stents). Total stent length was defined as the sum of lengths of all the stents implanted into the lesion site.

### 2.3. Clinical data and treatment outcomes

The baseline characteristics, angiographic and procedural profiles, and post-discharge treatment outcomes were evaluated through a retrospective review and analysis of the database from CNUH.

Baseline characteristics data included age, sex, utilization of emergency medical services, Killip functional class, body mass index, past medical history, smoking history, family history of premature ischemic heart disease, serum creatinine (Cr) level, peak troponin-I level, medications at hospital discharge, use of thrombolysis, LVEF, and final diagnosis. Information collected about discharge medication included aspirin, P2Y12 inhibitors, beta blockers, angiotensin-converting enzyme inhibitors or angiotensin receptor blockers, and statins. Among the angiographic profiles, IRA, presence of LMCA disease and multivessel CAD, ACC/AHA lesion classification, TIMI flow grade, and all QCA results were included. Procedural information included stent diameter, stent length, vascular access, the use of glycoprotein IIb/IIIa inhibitors (GPIs), thrombus aspiration, and intracoronary imaging guidance.

Clinical follow-up was conducted for approximately 12 months. For treatment estimates, the primary endpoint was defined as a major adverse cardiac and cerebrovascular event (MACCE), which was a composite of the following outcomes: all-cause death, non-fatal myocardial infarction (NFMI), any revascularization, cerebrovascular accident (CVA), rehospitalization, and stent thrombosis. The secondary endpoints included each component of MACCE, including all-cause death, cardiac/non-cardiac death, NFMI, any revascularization [repeated revascularization (PCI or coronary artery bypass graft) of any portion of the entire coronary vasculature], culprit-lesion-related revascularization (repeated revascularization of culprit lesion), CVA, rehospitalization (first-time hospitalization with the chief complaint of angina pectoris or heart failure), and stent thrombosis [a definite or probable stent thrombosis, as stated in the definitions of the Academic Research Consortium (10)]. The independent clinical event monitoring committee, consisting of independent interventional cardiologists, adjudicated all clinical events in this study.

### 2.4. Statistical analysis

Participants were classified into group A (patients who underwent PCI with SLS only) or group B (those who underwent PCI with overlapped DSS). The two groups were compared for baseline characteristics and treatment outcomes. For each parameter, continuous variables are described as the mean with standard deviation and were analyzed using the student's *t*-test or analysis of variance. Discrete variables are described as frequencies with percentages and were analyzed using Pearson's chi-square test and Fisher's two-by-two exact test. A *p*-value < 0.05 was considered statistically significant.

To reduce the effects of selection bias due to different backgrounds between groups, inverse probability of treatment weighting (IPTW) was applied to adjust for different results in these variables and examine whether the stenting method affected the incidence of each treatment outcome independently. In IPTW, a matching ratio of 1:1 was applied, and the propensity score was constructed with a total of 42 covariates, including age, sex, utilization of emergency medical services, Killip functional class, body mass index, past medical history, smoking history, family history of premature ischemic heart disease, serum Cr level, peak troponin-I level, medications at hospital discharge, use of thrombolysis, LVEF, final diagnosis, IRA, presence of LMCA disease and multivessel CAD, ACC/AHA lesion classification, TIMI flow grade, all QCA results, stent diameter, vascular access, the use of GPIs, thrombus aspiration, and intracoronary imaging guidance.

Multivariable logistic regression analysis was conducted to assess variables that were correlated with stenting with overlapped DSS. Univariable logistic regression analysis was initially performed using baseline covariates, except for stent diameter, stent length, and medications at hospital discharge. Thereafter, the variables with a *p*-value < 0.2 were rendered for entry in the backward stepwise conditional logistic regression analysis.

All data were analyzed using STATA version 15.0 (StataCorp, College Station, TX, United States) and SPSS version 25.0 (IBM Corp., Armonk, NY, United States).

### 2.5. Ethics statements

This study was conducted in accordance with the ethical standards of the World Medical Association's Declaration of Helsinki. The present study was approved by the Institutional Review Board of CNUH (IRB No. CNUH-2022-136). The need for informed consent was waived because of the retrospective study design.

## 3. Results

### 3.1. Baseline patient characteristics

**Figure 3:**
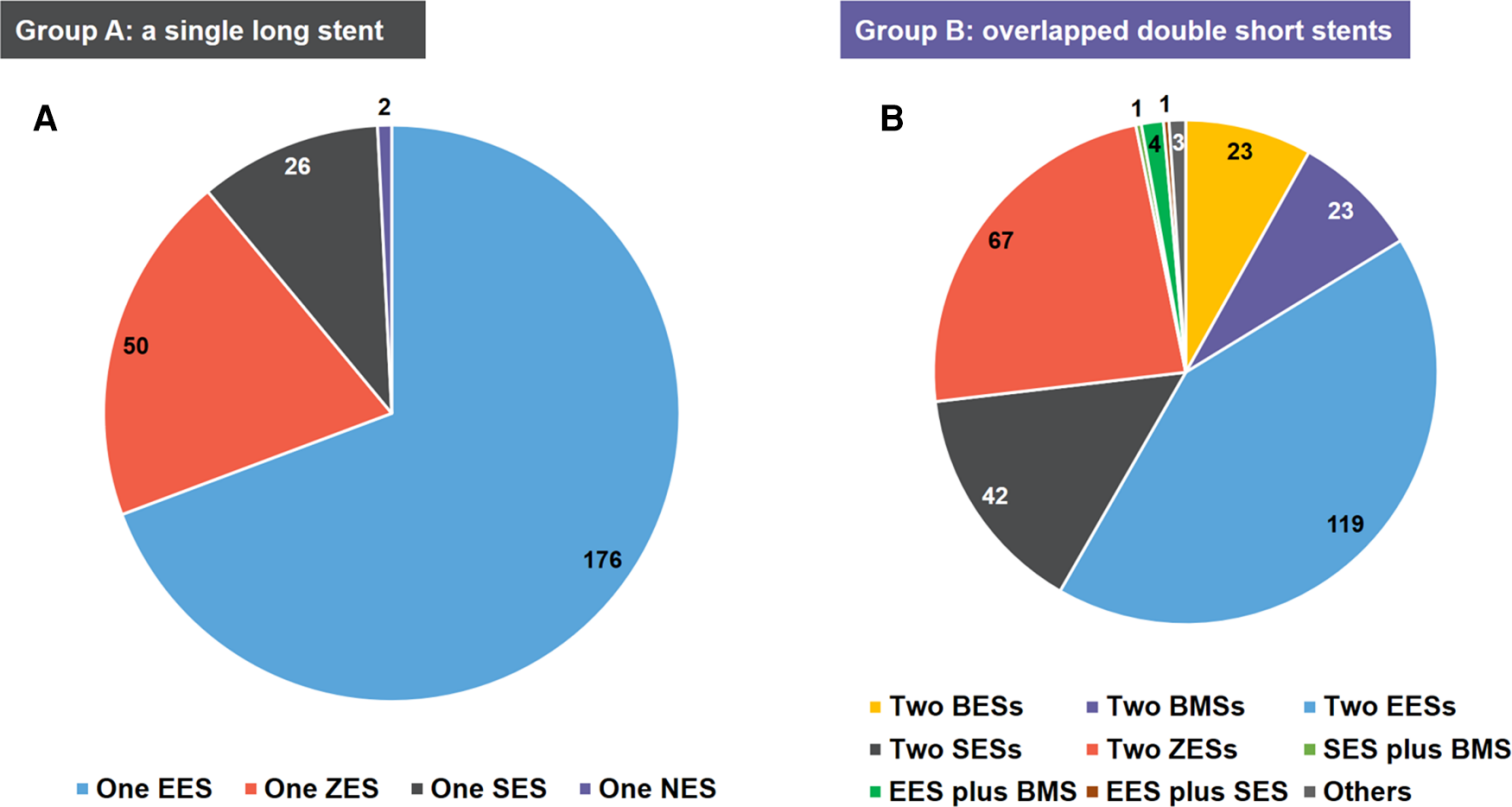
The distribution of stent types of the study population. (**A**) group A: a single long stent; (**B**) group B: overlapped double short stents. BES, biolimus A9-eluting stent; BMS, bare-metal stent; EES, everolimus-eluting stent; NES, novolimus-eluting stent; SES, sirolimus-eluting stent; ZES, zotarolimus-eluting stent.

A total of 537 patients with AMI who underwent stent implantation were included in the analysis. Of these, 254 (47.3%) patients were treated with an SLS and 283 (52.7%) with overlapped DSS. The distribution of stent types among the participants is detailed in Figure 3. In group A, the use of one everolimus-eluting stent was the most predominant type (*n* = 176), followed by one zotarolimus-eluting stent (*n* = 50), one sirolimus-eluting stent (*n* = 26), and one novolimus-eluting stent (*n* = 2). In group B, the use of two everolimus-eluting stents was the most predominant type (*n* = 119), followed by two zotarolimus-eluting stents (*n* = 67), two sirolimus-eluting stents (*n* = 42), and so forth.

Regarding baseline demographics and clinical characteristics (Table 1), most variables were comparable between the two groups, except for hypertension prevalence and serum Cr level. The prevalence of hypertension was higher in group B than in group A, whereas the rate of Cr ≥ 1.5 mg/dl was higher in group A than in group B. Angiographic and procedural profiles are summarized in Table 2. Compared with group A, group B had a greater total stent length. Group A had shorter total procedure time and lower total contrast volume than group B.

**Table 1:** Baseline demographic and clinical characteristics.

Characteristics	Before IPTW		*p*-value	After IPTW		*p*-value
	Group A	Group B		Group A	Group B	
	(*n* = 254)	(*n* = 283)		(*n* = 437)	(*n* = 449)	
Male patients, %	188 (74.0)	215 (76.0)	0.601	324 (74.2)	341 (75.9)	0.722
Age, years	64.63 ± 12.22	66.00 ± 12.63	0.202	64.38 ± 12.23	65.86 ± 12.02	0.249
Age ≥75 years, %	60 (23.6)	77 (27.2)	0.341	111 (25.3)	108 (24.1)	0.791
EMS utilization	20 (7.9)	20 (7.1)	0.722	20 (4.6)	23 (5.1)	0.804
Killip functional class III–IV, %	26 (10.3)	29 (10.2)	0.991	49 (11.1)	47 (10.6)	0.864
BMI, kg/m^2^	23.88 ± 3.28	23.96 ± 3.74	0.793	23.72 ± 3.42	23.81 ± 3.67	0.827
BMI ≥ 25 kg/m^2^, %	75 (33.5)	91 (34.9)	0.749	145 (33.3)	146 (32.4)	0.865
Past medical history						
Hypertension, %	119 (46.8)	158 (55.8)	**0**.**038**	220 (50.5)	227 (50.5)	0.997
Diabetes mellitus, %	86 (33.9)	86 (30.4)	0.390	140 (32.1)	140 (31.2)	0.865
Dyslipidemia, %	21 (8.3)	26 (9.2)	0.707	34 (7.8)	34 (7.5)	0.929
Prior IHD, %	34 (13.4)	36 (12.7)	0.819	63 (14.4)	62 (13.7)	0.851
Prior heart failure, %	5 (2.0)	6 (2.1)	0.901	8 (1.8)	8 (1.8)	0.970
Prior CVA, %	17 (6.7)	22 (7.8)	0.630	35 (8.1)	38 (8.4)	0.934
Smoking history, %	134 (53.2)	161 (57.1)	0.363	247 (56.7)	246 (54.9)	0.740
Family history of premature IHD, %	10 (4.1)	17 (6.1)	0.299	23 (5.4)	26 (5.9)	0.828
Serum Cr level	1.29 ± 1.51	1.08 ± 0.92	0.714	1.12 ± 1.01	1.25 ± 1.33	0.467
Cr ≥ 1.5 mg/dl, %	41 (16.3)	25 (8.9)	**0**.**009**	49 (11.3)	55 (12.2)	0.834
Peak troponin-I, ng/ml	41.64 ± 49.58	41.32 ± 49.75	**0**.**940**	39.77 ± 44.87	39.94 ± 46.59	0.970
Medications at discharge						
Aspirin, %	253 (99.6)	283 (100.0)	0.473	437 (100.0)	449 (100.0)	-
P2Y12 inhibitors, %	253 (99.6)	281 (99.3)	1.000	437 (100.0)	446 (99.3)	0.169
Beta-blockers, %	212 (83.5)	239 (84.5)	0.755	359 (82.2)	375 (83.6)	0.741
ACE inhibitors or ARBs, %	221 (87.0)	251 (88.7)	0.550	378 (86.6)	389 (86.6)	0.997
Statins, %	238 (93.7)	265 (93.6)	0.977	414 (95.0)	426 (94.8)	0.954
Use of thrombolysis, %	0 (0.0)	0 (0.0)	-	0 (0.0)	0 (0.0)	-
LVEF, %	53.17 ± 11.55	53.80 ± 11.03	0.523	52.42 ± 11.40	53.44 ± 11.15	0.411
LVEF < 40%, %	32 (13.3)	33 (11.8)	0.607	58 (13.2)	58 (12.8)	0.915
STEMI as a final diagnosis, %	118 (46.5)	127 (44.9)	0.714	213 (48.8)	219 (48.7)	0.975

Values are presented as number (percentage) for categorical values and means ± standard deviation for continuous variables. Bold values denote statistical significance at the *p*-value < 0.05 level.

ACE, angiotensin-converting enzyme; ARB, angiotensin receptor blocker; BMI, body mass index; Cr, creatinine; CVA, cerebrovascular accident; EMS, emergency medical service; IHD, ischemic heart disease; IPTW, inverse probability of treatment weighting; LVEF, left ventricular ejection fraction; STEMI, ST-segment elevation myocardial infarction.

**Table 2:** Angiographic and procedural profiles.

Characteristics	Before IPTW		*p*-value	After IPTW		*p*-value
	Group A	Group B		Group A	Group B	
	(*n* = 254)	(*n* = 283)		(*n* = 437)	(*n* = 449)	
Angiographic profiles						
Culprit vessel			0.929			0.947
LMCA, %	5 (2.0)	4 (1.4)		5 (1.2)	4 (1.0)	
LAD, %	140 (55.1)	162 (57.2)	** **	243 (55.7)	260 (57.8)	** **
LCX, %	29 (11.4)	32 (11.3)	** **	49 (11.2)	52 (11.6)	** **
RCA, %	80 (31.5)	85 (30.0)	** **	139 (31.9)	133 (29.5)	** **
LMCA disease, %	5 (2.0)	8 (2.8)	0.518	7 (1.7)	9 (2.1)	0.751
Multivessel CAD, %	77 (30.3)	79 (27.9)	0.541	123 (28.1)	120 (26.7)	0.780
ACC/AHA lesion classification			0.930			0.619
A/B1 lesion, %	6 (2.4)	7 (2.5)	** **	13 (3.0)	9 (2.1)	
B2/C lesion, %	244 (97.6)	271 (97.5)	** **	423 (97.0)	440 (97.9)	
Preprocedural TIMI flow grade 0-I, %	137 (54.4)	136 (48.7)	0.196	233 (53.4)	227 (50.5)	0.599
QCA results						
Lesion length, mm	38.04 ± 2.43	38.54 ± 9.31	0.376	37.87 ± 2.06	38.07 ± 9.16	0.772
Mean RVD, mm	2.98 ± 0.37	3.01 ± 0.44	0.335	3.00 ± 0.36	2.99 ± 0.42	0.918
Proximal RVD, mm	3.04 ± 0.35	3.07 ± 0.45	0.293	3.06 ± 0.34	3.05 ± 0.43	0.885
Distal RVD, mm	2.81 ± 0.42	2.81 ± 0.48	0.951	2.79 ± 0.52	2.80 ± 0.45	0.969
MLD, mm	0.20 ± 0.29	0.68 ± 6.17	0.214	0.23 ± 0.31	0.23 ± 0.33	0.999
Percent diameter stenosis, %	93.16 ± 10.30	77.86 ± 10.30	0.193	92.14 ± 11.53	92.15 ± 11.52	0.994
Procedural profiles						
Mean stent diameter, mm	3.03 ± 0.36	3.07 ± 0.42	0.216	3.05 ± 0.34	3.05 ± 0.40	0.915
Total stent length, mm	38.26 ± 1.31	45.20 ± 9.25	**<0**.**001**	38.28 ± 1.47	44.63 ± 9.04	**<0**.**001**
Femoral approach, %	101 (39.8)	112 (39.6)	0.965	178 (40.7)	182 (40.6)	0.973
Use of glycoprotein IIb/IIIa inhibitors, %	29 (11.4)	47 (16.6)	0.085	64 (14.8)	71 (15.8)	0.778
Use of thrombus aspiration, %	26 (10.2)	37 (13.1)	0.308	65 (14.8)	61 (13.6)	0.769
Intracoronary imaging guidance, %	12 (4.7)	23 (8.1)	0.111	20 (4.6)	27 (6.1)	0.502
Total procedure time, min	41.40 ± 15.74	53.31 ± 21.75	**<0**.**001**	45.36 ± 16.57	47.67 ± 20.51	0.265
Total contrast volume, ml	134.13 ± 30.72	160.57 ± 39.77	**<0**.**001**	145.69 ± 33.40	149.78 ± 36.24	0.295

Values are presented as number (percentage) for categorical values and means ± standard deviation for continuous variables. Bold values denote statistical significance at the *p*-value < 0.05 level.

ACC/AHA, the American College of Cardiology/the American Heart Association; CAD, coronary artery disease; IPTW, inverse probability of treatment weighting; MLD, minimum lumen diameter; LAD, left anterior descending coronary artery; LCX, left circumflex coronary artery; LMCA, left main coronary artery; QCA, quantitative coronary angiography; RCA, right coronary artery; RVD, reference vessel diameter; TIMI, Thrombolysis In Myocardial Infarction.

After IPTW, the different trends in all covariates of baseline clinical and procedural characteristics were adequately balanced between groups (Tables 1, 2).

### 3.2. Treatment outcomes

**Figure 4:**
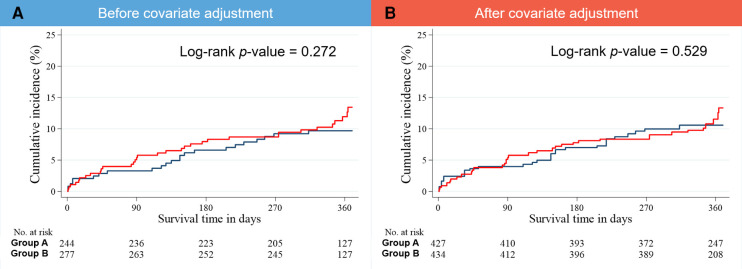
Kaplan–Meier estimates of the cumulative incidence of 1-year MACCE (**A**) before and (**B**) after covariate adjustment. MACCE, major adverse cardiac and cerebrovascular event.

The median follow-up interval was 364 days. Treatment outcomes during the 1-year follow-up, including MACCE and its individual components (all-cause death, cardiac death, noncardiac death, NFMI, any revascularization, culprit-lesion-related revascularization, CVA, rehospitalization, and stent thrombosis) were recorded (Table 3; Figure 4). Comparable outcomes were observed between groups. Regarding the IPTW-adjusted data, no significant difference between groups was evident for any treatment outcome.

**Table 3:** Post-discharge treatment outcomes of patients who were successfully discharged with PCI.

Treatment outcomes	Event rates		Unadjusted analysis		IPTW-adjusted analysis	
	Group A (*n* = 244)	Group B (*n* = 277)	1-year HR (95% CI)	*p*-value	1-year HR (95% CI)	*p*-value
MACCE	24 (9.8)	37 (13.4)	1.33 (0.80–2.24)	0.274	1.21 (0.67–2.19)	0.529
All-cause death	9 (3.7)	17 (6.1)	1.68 (0.75–3.76)	0.211	1.74 (0.70–4.31)	0.235
Cardiac death	7 (2.9)	13 (4.7)	1.65 (0.66–4.13)	0.286	2.28 (0.85–6.11)	0.102
Non-cardiac death	2 (0.8)	4 (1.4)	1.77 (0.32–9.67)	0.510	0.94 (0.16–5.52)	0.947
NFMI	7 (2.9)	5 (1.8)	0.63 (0.20–1.99)	0.435	0.53 (0.15–1.86)	0.324
Any revascularization	10 (4.1)	14 (5.0)	1.17 (0.51–2.67)	0.710	1.05 (0.41–2.67)	0.926
Culprit-lesion-related revascularization	5 (2.0)	5 (1.8)	0.72 (0.19–2.67)	0.621	0.53 (0.12–2.35)	0.406
CVA	2 (0.8)	2 (0.7)	0.88 (0.12–6.25)	0.899	2.35 (0.21–26.98)	0.491
Rehospitalization	8 (3.3)	5 (1.8)	0.56 (0.18–1.70)	0.302	0.44 (0.13–1.44)	0.173
Stent thrombosis	1 (0.4)	0 (0.0)	–	–	–	–

Values are presented as percentage (number) for categorical values.

CI, confidence interval; CVA, cerebrovascular accident; HR, hazard ratio; IPTW, inverse probability of treatment weighting; MACCE, major adverse cardiac and cerebrovascular events; NFMI, non-fatal myocardial infarction; PCI, percutaneous coronary intervention; ST, stent thrombosis.

### 3.3. Independent factors for stenting with overlapped DSS

When assessing correlates of stenting with overlapped DSS during PCI using multivariable logistic regression analysis, it was shown that hypertension [adjusted odds ratio (OR), 2.16; 95% confidence interval (CI), 1.39–3.36], serum Cr level ≥1.5 mg/dl (adjusted OR, 0.37; 95% CI, 0.19–0.74), and the use of GPIs (adjusted OR, 2.09; 95% CI, 1.09–3.99) were strongly associated with this stenting method (Table 4).

**Table 4:** Independent factors for stenting with overlapped DSS.

	Univariable analysis		Multivariable analysis	
	OR (95% CI)	*p*-value	OR (95% CI)	*p*-value
Sex (male)	1.11 (0.75–1.64)	0.601	1.31 (0.74–2.32)	0.352
Age ≥75 years	1.21 (0.82–1.79)	0.341	1.24 (0.74–2.07)	0.415
EMS utilization	0.89 (0.47–1.69)	0.722	1.96 (0.75–5.07)	0.167
Killip functional class III–IV	1.00 (0.57–1.74)	0.991	1.47 (0.72–2.99)	0.286
BMI ≥ 25 kg/m^2^	1.06 (0.73–1.55)	0.749	0.95 (0.61–1.49)	0.839
Hypertension	1.43 (1.02–2.02)	0.038	2.16 (1.39–3.36)	**0**.**001**
Diabetes mellitus	0.85 (0.59–1.23)	0.390	0.78 (0.49–1.23)	0.281
Dyslipidemia	1.12 (0.61–2.05)	0.707	1.68 (0.75–3.78)	0.207
Prior IHD	0.94 (0.57–1.56)	0.819	0.78 (0.43–1.41)	0.415
Prior heart failure	1.08 (0.33–3.58)	0.901	0.85 (0.21–3.44)	0.816
Prior CVA	1.18 (0.61–2.27)	0.630	1.00 (0.47–2.16)	0.990
Smoking history	1.17 (0.83–1.65)	0.364	1.04 (0.64–1.68)	0.871
Family history of premature IHD	1.52 (0.68–3.40)	0.302	1.47 (0.61–3.54)	0.390
Cr ≥ 1.5 mg/dl	0.50 (0.29–0.85)	0.010	0.37 (0.19–0.74)	**0**.**005**
Peak troponin-I, ng/ml	1.00 (0.99–1.00)	0.940	1.00 (1.00–1.01)	0.490
LVEF <4 0%	0.87 (0.52–1.47)	0.607	1.41 (0.73–2.75)	0.308
STEMI as a final diagnosis	0.94 (0.67–1.32)	0.714	0.96 (0.59–1.56)	0.862
Culprit vessel				
LMCA	1 (reference)		1 (reference)	
LAD	1.45 (0.38–5.49)	0.588	9.94 (0.66–149.74)	0.097
LCX	1.38 (0.34–5.64)	0.654	10.39 (0.64–167.63)	0.099
RCA	1.33 (0.34–5.12)	0.680	9.28 (0.59–147.14)	0.114
LMCA disease	1.45 (0.47–4.49)	0.520	4.66 (0.48–45.64)	0.186
Multivessel CAD	0.89 (0.61–1.29)	0.541	0.92 (0.57–1.49)	0.740
B2/C lesion as ACC/AHA lesion classification	0.95 (0.32–2.87)	0.930	0.93 (0.24–3.56)	0.912
Preprocedural TIMI flow grade 0-I	0.80 (0.57–1.12)	0.196	0.72 (0.44–1.18)	0.187
Lesion length, mm	1.01 (0.99–1.04)	0.398	1.02 (0.99–1.06)	0.141
Mean RVD, mm	1.22 (0.81–1.85)	0.339	5.55 (0.65–47.18)	0.117
Proximal RVD, mm	1.25 (0.82–1.90)	0.299	0.69 (0.15–3.16)	0.628
Distal RVD, mm	0.99 (0.68–1.44)	0.951	0.29 (0.08–1.10)	0.069
MLD, mm	1.73 (1.01–2.97)	0.047	0.53 (0.01–20.57)	0.734
Percent diameter stenosis, %	0.98 (0.97–1.00)	0.052	0.96 (0.87–1.07)	0.511
Femoral approach	0.99 (0.70–1.40)	0.965	0.99 (0.63–1.55)	0.957
Use of glycoprotein IIb/IIIa inhibitors	1.55 (0.94–2.54)	0.087	2.09 (1.09–3.99)	**0**.**026**
Use of thrombus aspiration	1.32 (0.77–2.25)	0.309	0.99 (0.49–2.01)	0.988
Intracoronary imaging guidance	1.78 (0.87–3.66)	0.115	2.16 (0.86–5.41)	0.102

Bold values denote statistical significance at the *p*-value < 0.05 level.

ACC/AHA, American College of Cardiology/American Heart Association; BMI, body mass index; CAD, coronary artery disease; CI, confidence interval; Cr, creatinine; DSS, double short stents; EMS, emergency medical service; IHD, ischemic heart disease; LAD, left anterior descending coronary artery; LCX, left circumflex coronary artery; LMCA, left main coronary artery; LVEF, left ventricular ejection fraction; MLD, minimum lumen diameter; OR, odds ratio; RCA, right coronary artery; RVD, reference vessel diameter; STEMI, ST-segment elevation myocardial infarction; TIMI, thrombolysis in myocardial infarction.

## 4. Discussion

The present study utilized clinical information from patients with AMI treated with either an SLS or overlapped DSS from a single-center database and evaluated 1-year treatment outcomes. The main findings of the present study are that SLS provides 1-year treatment outcomes comparable with those in overlapped DSS in patients with AMI.

Since the first report of balloon angioplasty in 1977, the PCI procedure has markedly evolved, changing reperfusion practices for patients with CAD. Because coronary artery stenting becomes widely used, the chance of encountering and treating complex coronary lesions in real-world PCI practices has increased. The diffuse CAD is more frequently encountered (1, 11); therefore, many interventional cardiologists are occasionally forced to choose interventional strategies, including the single-stent technique with an SLS or the overlapping stent technique (OST) with DSS, to cover diffuse long-length coronary lesions (11).

OST accounts for up to 30% of PCI due to extensive lesion length, stent edge dissections, or incomplete stent coverage (6, 12–14). In the bare-metal stent (BMS) era, OST was associated with increased target lesion revascularization rates compared with those of the single-stent technique (6). However, with the advent of first-generation DESs, clinical and angiographic restenosis were markedly declined with the potent suppression of neointimal hyperplasia (15, 16). Furthermore, OST demonstrated improved and acceptable safety and efficacy in several DES-based studies (17, 18), leading to its wider application. Despite this revolutionary advancement, potential concerns with OST remain (11). First, OST increases the risk both of side branch compromise and periprocedural myonecrosis from the stent struts double layer or plaque shifting (19, 20). Second, OST forms an inadvertent gap between the stents, increasing the risk of acute or subacute stent thrombosis and late restenosis at this site (11). Third, while overlapping more stent platforms may minimize the stented area in complex calcified lesion, it may also increase the chances of more malapposed or more damaged struts in lesions where stent advancement is made with difficulty (6, 21, 22). Moreover, stent strut fractures can occur in overlapping zones, especially in the presence of coronary arterial curvature (23). Finally, OST increases the risk of repeat revascularization or adverse ischemic events (6). According to two DES-based large-scale clinical studies: the SIRTAX (Sirolimus-Eluting Vs. Paclitaxel-Eluting Stents for Coronary Revascularization) trial and LESSON (Long-term comparison of Everolimus-eluting and Sirolimus-eluting Stents for cOronary revascularizatioN) registry (5, 6), OST demonstrated worse clinical outcomes with more prevalent adverse cardiac events; nonetheless, these findings were not evident among patients treated using only second-generation DESs.

Based on these factors and clinical evidence, the clinical outcomes of multiple overlapping DESs remain debatable, even in the DES era. As such, the use of one long stent, rather than two short stents, may be preferable, given its appropriate implementation. Therefore, we compared these two stenting methods in patients with AMI undergoing PCI. In this study, implantations of SLS and DSS were performed in participants with similar clinical severity of their conditions, except for a few variables. Group A had worse kidney function with a higher proportion of Cr ≥ 1.5 mg/dl, a lower prevalence of hypertension, and shorter stent length than group B. Despite these differences, stenting with SLS showed a similar incidence of MACCEs compared to its counterpart; these trends were maintained even after IPTW adjustment. Also, considering that our ‘real-world’ data included a total of 28 cases with any BMS, and it is widely accepted that BMS has higher rates of restenosis than DES, a statistical analysis excluding those with any BMS was additionally conducted, demonstrating consistent results (Supplementary Table S1).

Based on the literature review, the outcomes shown in some previous clinical studies are consistent with those of the current study. Mori and colleagues showed that everolimus-eluting stents had similar angiographic and 1-year follow-up outcomes between SLS and overlapped DSS (24). Moreover, Jurado-Román et al. compared clinical outcomes of long stents and overlapped stents in diffuse CAD, emphasizing some advantages of PCI with those of long stents (1). Yano et al. demonstrated that long DES implantation had both acceptable and comparable outcomes for up to 2 years after PCI (25), and Sim et al. presented similar results (26). One meta-analysis also demonstrated that the use of SLS showed lower rates of cardiac death and target lesion revascularization than that of two or more short stents (27). Despite these prior studies, there is insufficient real-world clinical evidence concerning comparative treatment outcomes of these two stenting techniques in AMI settings. Since the present study only included patients with AMI mostly treated by second-generation DESs, our results underscore that SLS shows comparable outcomes to those of DSS, even in the clinical setting of AMI—the most severe form of CAD.

Despite similarities in treatment outcomes, PCI with SLS may have more potential treatment advantages than that with DSS. First, treatment is more economical and efficient because fewer stents are required. Second, very long stents may simplify the procedure, reducing total procedure time, fluoroscopy time, radiation exposure, and the amount of contrast media. Regarding Jurado-Román et al.'s study (1), PCI with long stents had lower contrast volume, shorter procedure duration, and shorter fluoroscopy time than that with multiple shorter stents, which may align with our results demonstrating that group A had shorter total procedure time but lower contrast volume than group B. Third, SLS can prevent potential complications due to stent overlapping. Considering these aspects, SLS appears to be a good choice for PCI in this population.

Three independent factors for stenting with DSS during PCI were identified. Interestingly, both hypertension and the use of GPIs were positive factors, whereas serum Cr was a negative factor. Given that impaired kidney function is associated with poor peri-procedural outcomes during PCI (28), it is reasonable to choose the SLS method for simplicity. Conversely, it is somewhat difficult to clarify why both hypertension and the use of GPIs were independently associated with stenting with DSS. Pre-existing hypertension is a well-established risk factor for atherosclerotic cardiovascular diseases and is associated with diffuse atherosclerosis (29). According to one clinical study, hypertension seemingly has the potential to aggravate the extent and severity of CAD, although this effect was limited to patients with diabetes. Therefore, it is plausible that hypertension may induce diffuse CAD (30), having extended atherosclerotic plaques, thereby increasing the requirement for OST with DSS. Meanwhile, since GPIs are effective on lowering thrombus burden (31), they have been shown to provide clinical benefits in high-risk patients undergoing PCI (32–34), their use is recommended in cases of a high thrombus burden to minimize the risk of the no-reflow phenomenon (35, 36). Given that stent overlapping is related to delayed arterial healing and increased inflammation (6, 37), it is plausible that OST may also promote intracoronary thrombus formation (38, 39), consequently requiring downstream administration of GPI. Of course, since these explanations are speculative at present, further investigation is required to elucidate this association.

Besides our analysis demonstrated that the rates of intracoronary imaging guidance were very low (4.7% in group A, and 8.1% in group B). Despite the fact that both IVUS and OCT are well-established tools to guide and optimize PCI (40), and their use for PCI is rapidly increasing (41, 42), they are used in a small proportion of all PCI in the setting of AMI (41, 42). According to 2023 European Society of Cardiology guidelines on acute coronary syndromes, both of them should be considered for culprit lesions (Class IIa), and may also be considered for non-culprit lesions (Class IIb) (40). Hence, if they become more widely used, it may help achieve optimized stent expansion with more acceptable values of post-PCI minimum stent area, which may reduce underexpansion-related complications and demonstrate more acceptable outcomes, especially in OST with DSS (43, 44). Since these strengths are necessary in clinical situations that require relatively sophisticated stenting techniques, their utilization may contribute to minimizing OST-related complications mentioned earlier.

### 4.1. Study limitations

Although similar outcomes for both SLS and DSS in PCI in patients with AMI were observed, our results must be interpreted with caution owing to several methodological limitations. First, this study was a non-randomized retrospective analysis of a single-center database. Second, although covariate adjustment was conducted to minimize selection bias, it may have remained due to several reasons, such as inclusion and exclusion criteria, intentional exclusion of data in cases of missing information, and other potential unmeasured confounders. Third, establishing causation between the stenting method and each treatment estimate was difficult due to the non-randomized and retrospective nature of the study. Fourth, while many previous studies reported more detailed information on lesion characteristics (5, 6, 26), some detailed angiographic information including tortuosity, eccentricity, angulation, vessel diameter variability, presence of side branches, and degree of coronary artery calcification, was missing from the present study. Considering these limitations, the results may not be generalizable but must be interpreted as hypothesis-generating.

## 5. Conclusions

In this study, we evaluated the baseline characteristics and treatment outcomes of an SLS vs. overlapped DSS in the clinical setting of AMI. We demonstrated that stenting with an SLS produced comparable outcomes to those of stenting with DSS. Some demographic and clinical conditions seem to be independently associated with these stenting methods. Given the more potential treatment advantages with SLS, if its deliverability is acceptable, stenting with an SLS may be a safe and effective treatment strategy for patients with AMI, as opposed to that with DSS.

## Data Availability

The raw data supporting the conclusions of this article will be made available by the authors, without undue reservation.
