# Supplementary material for: Comparative treatment outcomes of a single long stent vs. overlapped short stents in acute myocardial infarction
Source: Front Cardiovasc Med. 2023 Dec 19;10:1284396. doi: 10.3389/fcvm.2023.1284396 (PMC10766367; doi:10.3389/fcvm.2023.1284396)
Supplement: Supplementary file 1 [file Table1.docx]

**Supplemental Table 1.** Treatment Outcomes Excluding Cases with Any BMS

| Treatment outcomes | Event rates | | p-value |
| --- | --- | --- | --- |
|  | **Group A** | **Group B** |  |
|  | **(n = 244)** | **(n = 250)** |  |
| MACCE | 24 (9.8) | 27 (10.8) | 0.725 |
| All-cause death | 9 (3.7) | 9 (3.6) | 0.958 |
| Cardiac death | 7 (2.9) | 8 (3.2) | 0.830 |
| Non-cardiac death | 2 (0.8) | 1 (0.4) | 0.620 |
| NFMI | 7 (2.9) | 4 (1.6) | 0.377 |
| Any revascularization | 10 (4.1) | 13 (5.2) | 0.561 |
| Culprit-lesion-related revascularization | 5 (2.0) | 4 (1.6) | 0.749 |
| CVA | 2 (0.8) | 2 (0.8) | 1.000 |
| Rehospitalization | 8 (3.3) | 4 (1.6) | 0.226 |
| Stent thrombosis | 1 (0.4) | 0 (0.0) | 0.494 |

Values are presented as percentage (number) for categorical values.

CVA, cerebrovascular accident; MACCE, major adverse cardiac and cerebrovascular events; NFMI, non-fatal myocardial infarction.
